# Correction to “The Modernized Traditional Diet: A Major Risk Factor for Hypertension and Multimorbidity in Belize's Multiethnic Population”

**DOI:** 10.1002/fsn3.72406

**Published:** 2026-09-23

Husaini, D. C. , L. J. Johnson , and C. Nwokocha . 2026. “The Modernized Traditional Diet: A Major Risk Factor for Hypertension and Multimorbidity in Belize's Multiethnic Population.” Food Science & Nutrition 14, no. 6: e71986. 10.1002/fsn3.71986.

In Section 2.2.1, the estimated population in the sentence “Belize has a small, dispersed adult population (estimated ~285,000)” was incorrect. It should read: “Belize has a small, dispersed adult population (estimated ~420,000).”

We apologize for this error.

